# A Novel Overall Survival Prediction Signature Based on Cancer Stem Cell-Related Genes in Osteosarcoma

**DOI:** 10.3389/fcell.2021.753414

**Published:** 2021-10-18

**Authors:** Bo Xiao, Liyan Liu, Zhuoyuan Chen, Aoyu Li, Yu Xia, Pingxiao Wang, Cheng Xiang, Yi Zeng, Hui Li

**Affiliations:** ^1^Department of Orthopedics, The Second Xiangya Hospital, Central South University, Changsha, China; ^2^Orthopedic Biomedical Materials Engineering Laboratory of Hunan Province, Changsha, China

**Keywords:** osteosarcoma, stem cells, gene, overall survival, prognosis, signature

## Abstract

**Background:** Osteosarcoma is the most general bone malignancy that mostly affects children and adolescents. Numerous stem cell-related genes have been founded in distinct forms of cancer. This study aimed at identifying a stem cell-related gene model for the expected assessment of the prognosis of osteosarcoma patients.

**Methods:** We obtained the genes expression data and relevant clinical materials from Therapeutically Applicable Research to Generate Effective Treatments (TARGET) and Gene Expression Omnibus (GEO) databases. We identified differentially expressed genes (DEGs) from the GEO dataset, whereas prognostic stem cell-related genes were obtained from the TARGET database. Subsequently, univariate, LASSO and multivariate Cox regression analyses were applied to establish the stem cell-related signature. Finally, the prognostic value of the signature was validated in the GEO dataset.

**Results:** Twenty-five genes were prognostic ferroptosis-related DEGs. Consequently, we identified eight stem cell-related genes as a signature of prognosis of osteosarcoma patients. Then, the Kaplan–Meier (K-M) curve, the AUC value of ROC, and Cox regression analysis verified that the eight stem cell-related gene model were a new and substantial prognostic marker independent of other clinical traits. Moreover, the nomogram on the foundation of risk score and other clinical traits was established for predicting the survival rate of osteosarcoma patients. Biological function analyses displayed that tumor related pathways were affluent.

**Conclusion:** The expression level of stem cell-related genes offers novel prognostic markers as well as underlying therapeutic targets for the therapy and prevention of osteosarcoma.

## Background

Osteosarcoma (OS) is the most widespread main bone malignancy in children and young adults (Rothzerg et al., 2020) as well as makes up for nearly two-thirds of the primary bone malignancies diagnosed in the first 20 years of one’s life (Yan et al., 2016; Chen et al., 2019). The combination of surgical resection skills and multiagent chemotherapy has extremely enhanced the prognosis of osteosarcoma patients (Bielack et al., 2002). Other studies have shown that over the last 30 years, neoadjuvant chemotherapy and surgical techniques have developed, and the survival rate of 5 years in these patients has increased to ∼70% (Bielack et al., 2002; Niu et al., 2019; Wakamatsu et al., 2019). On the other hand, the overall survival rate of 5 years in cases diagnosed with early pulmonary metastasis is less than 20%. Besides, another study shows that 20% of patients without early pulmonary metastasis have local recurrence or will eventually develop pulmonary metastasis (Bishop et al., 2016). However, the fact that these patients develop resistance to chemotherapy is a major challenge. Therefore, it is imperative to explore novel prognostic models to further improve the survival of osteosarcoma patients.

Cancer stem cells (CSCs), a small, special class of cells inside tumors, owns the capability of self-renewal and differentiation of different cell types that assemble the whole tumor (Reya et al., 2001). CSCs are tumor-derived cells that mutate from stem cells of adults and produce distinct subtypes of tumor cells via self-renewal and differentiation (van der Heijden and Vermeulen, 2019). CSCs were associated with the development, recurrence, metastasis, and resistance of cancers and facilitate tumor progression (Dawood et al., 2014; Nassar and Blanpain, 2016). The capability of CSCs to originate and maintain the proliferation of cancer cells is an essential factor for metastasis. CSCs are heterogeneous and own subgroups with metastatic ability (Nandy and Lakshmanaswamy, 2017; Aydemir Coban and Sahin, 2018). Besides, CSCs can stay in prolonged dormancy and have varieties of molecular mechanisms of chemotherapy resistance (Lytle et al., 2018). As CSCs act as crucial roles in tumor progression, CSC biomarkers and related pathways may become the prognostic biomarkers and underlying targets for treatment of osteosarcoma patients.

Herein, we mined the expression data of genes and the relevant clinical information of osteosarcoma samples, and CSC-related genes from the opening databases. Next, the prognostic CSC-related gene signature was built in the Therapeutically Applicable Research to Generate Effective Treatments (TARGET) cohort and then evaluated it in the Gene Expression Omnibus (GEO) dataset. Last, we explored the potential mechanisms underlying CSC-related genes using a functional enrichment analysis.

## Materials and Methods

### Data Collection

The TARGET database was applied to acquire the mRNA expression data and corresponding clinical features of osteosarcoma samples. The information of clinical follow-up in this cohort is shown in Table 1.

**TABLE 1:** Clinical features of the osteosarcoma samples in the Therapeutically Applicable Research to Generate Effective Treatments (TARGET) and Gene Expression Omnibus (GEO) databases.

**Variables**	**TARGET (*n*)**	**GEO (*n*)**	
**Age (year)**			
≤16	56	25	
>16	39	28	
**Gender**			
Female	40	19	
Male	55	34	
**Race**		**Huvos grade**	
White	55	1	13
Asian	6	2	16
Black	9	3	13
Other	1	4	5
Unknown	24	Unknown	6
**Metastasis**			
Yes	23	34	
No	72	19	
**Survival status**			
Alive	57	30	
Death	38	23	
**Primary tumor sites**			
Leg/foot	83	44	
Arm/hand	7	8	
Pelvis	4	/	
Other/unknown	1	1	

The mRNA expression of GSE33383 and GSE21257 were mined from the GEO database; the package of “limma” was applied for quality control and normalization. The platform of GSE33383 and GSE21257 is GPL10295. The expression value for genes with numerous probes was computed as the average of the probes. The dataset of GSE33383 contains 15 mesenchymal stem cells/osteoblasts and 84 samples of osteosarcoma tissues, while the dataset of GSE21257 contains 53 patients of osteosarcoma, serving as a validating set. The clinical features of GSE21257 are shown in Table 1.

The CSC-related pathways were mined from the Molecular Signature Database v 7.4 (MSigDB). Altogether, 413 CSC-related genes were classified from the 31 pathways in the Gene Ontology (GO) and reactome databases (Table 2).

**TABLE 2:** Pathways associated with cancer stem cells in the Gene Ontology (GO) and reactome databases.

**Stem cell function related pathways**	**ID**	**Gene number**
GO: Stem cell division	GO:0017145	25
GO: Somatic stem cell population maintenance	GO:0035019	71
GO: Hematopoietic stem cell migration	GO:0035701	8
GO: Somatic stem cell division	GO:0048103	9
GO: Stem cell differentiation	GO:0048863	251
GO: Stem cell fate commitment	GO:0048865	6
GO: Hematopoietic stem cell differentiation	GO:0060218	88
GO: Hematopoietic stem cell homeostasis	GO:0061484	15
GO: Hematopoietic stem cell proliferation	GO:0071425	25
GO: Mesenchymal stem cell maintenance involved in nephron morphogenesis	GO:0072038	5
GO: Stem cell proliferation	GO:0072089	64
GO: Regulation of stem cell proliferation	GO:0072091	41
GO: Mesenchymal stem cell differentiation	GO:0072497	9
GO: Neuronal stem cell population maintenance	GO:0097150	19
GO: Mesenchymal stem cell proliferation	GO:0097168	8
GO: Regulation of hematopoietic stem cell proliferation	GO:1902033	11
GO: Positive regulation of hematopoietic stem cell proliferation	GO:1902035	6
GO: Regulation of hematopoietic stem cell differentiation	GO:1902036	74
GO: Negative regulation of hematopoietic stem cell differentiation	GO:1902037	5
GO: Negative regulation of stem cell population maintenance	GO:1902455	7
GO: Positive regulation of stem cell population maintenance	GO:1902459	7
GO: Positive regulation of mesenchymal stem cell proliferation	GO:1902462	5
GO: Regulation of somatic stem cell population maintenance	GO:1904672	8
GO: Regulation of stem cell division	GO:2000035	10
GO: Regulation of stem cell population maintenance	GO:2000036	31
GO: Negative regulation of stem cell proliferation	GO:2000647	9
GO: Positive regulation of stem cell proliferation	GO:2000648	22
GO: Regulation of stem cell differentiation	GO:2000736	112
GO: Negative regulation of stem cell differentiation	GO:2000737	21
GO: Positive regulation of stem cell differentiation	GO:2000738	18
GO: Regulation of mesenchymal stem cell differentiation	GO:2000739	6
Reactome transcriptional Regulation of Pluripotent Stem Cells	R-HSA-452723	31

### Establishment of the Cancer Stem Cell-Related Gene Model in the Therapeutically Applicable Research to Generate Effective Treatments Database

To explore the differentially expressed genes (DEGs) among normal and osteosarcoma samples with the FDR value < 0.05, we employed the “limma” R package present in the GSE33383 cohort. Next, we employed univariate Cox analysis to compute the prognostic values of CSC-related genes (*p* < 0.05) for the DEGs in the TARGET database (Rothzerg et al., 2021). Then, the LASSO regression analysis was applied for screening the survival-related genes for the outcomes of univariate Cox regression analysis utilizing the “glmnet” package. Finally, to optimize the model, we applied the multivariate regression analysis, which constructs the prognostic signature on the foundation of the expression and corresponding coefficients of CSC-related genes. The formula of risk score was as follows: risk score = Σ*i*coefficient (gene*i*) × expression (gene*i*). Consequently, the median value of risk score was obtained employing the “Survminer” package. Here, the patients of osteosarcoma were classified into two risk groups (low and high). Moreover, the Kaplan–Meier (K-M) curve and time-dependent receiver-operating characteristic (ROC) curve were applied to assess the clinical prognostic risk score capacity. Furthermore, the curves of survival score and survival status as well as heatmap were employed to illustrate the distribution of osteosarcoma patients in two groups (high risk and low risk). Last, we used different variables such as gender, age, tumor site, risk score, and metastasis to evaluate whether the risk score is an independent prognostic biomarker for patients of osteosarcoma, using uni- and multivariate Cox regression analysis.

### Establishment and Calibration of Nomogram

A nomogram was employed for predicting the survival of osteosarcoma patients. Here, different variables such as age, gender, risk score, race, tumor site, and metastasis were employed to construct the nomogram utilizing the R packages “rms” and “survival.” Finally, the resulting calibration curve was applied to assess the accurateness of the nomogram between different groups of patients.

### Functional Enrichment Analysis

The GO that comprises biological processes (BP), cellular components (CC), and molecular functions (MF), as well as KEGG analyses were achieved in two risk groups (low and high) utilizing the “clusterProfiler” R package with | log2FC| > 1 and FDR value < 0.05.

### Statistical Analysis

Here, the DEGs among normal and osteosarcoma cohorts were calculated using the Wilcox test. The K-M curve was employed to compare the overall survival between the two risk groups using the Chi-square test. Subsequently, Cox regression analyses were carried out to study independent prognostic variables. The R statistical software (Version 4.0.1) was employed to execute all statistical analyses.

## Results

**FIGURE 1:**
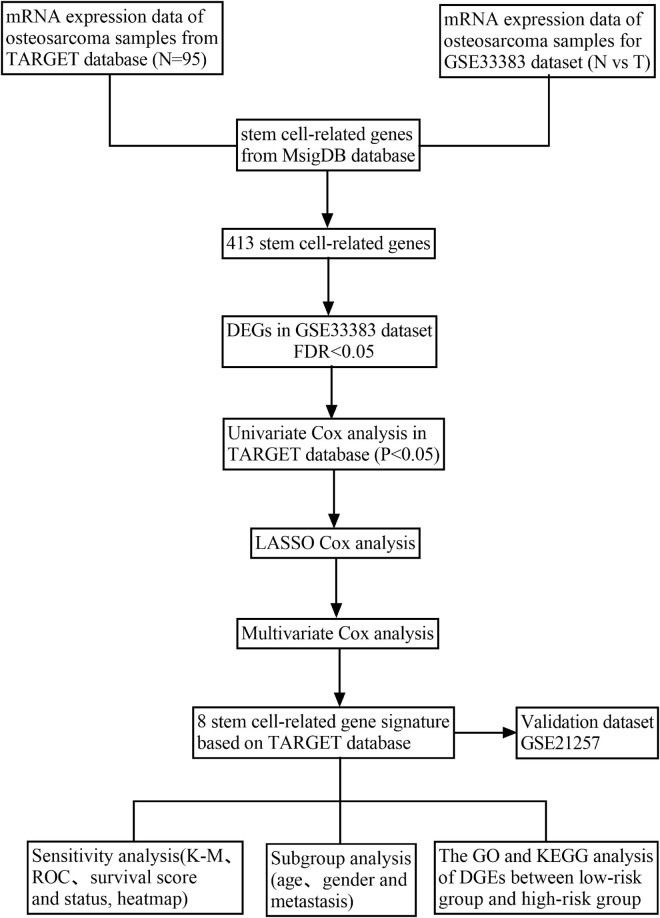
Flow chart of data gathering and analysis.

Figure 1 demonstrates a flow chart that explains the study. In this study, we mined data for a total of 95 patients with osteosarcoma and who had complete clinical information from the TARGET database, 15 normal samples, and 84 osteosarcoma samples in GSE33383 from the GEO database. Besides, the GSE21257 served as the validating cohort.

### Distinction of Prognostic Cancer Stem Cell-Related Differentially Expressed Genes

**FIGURE 2:**
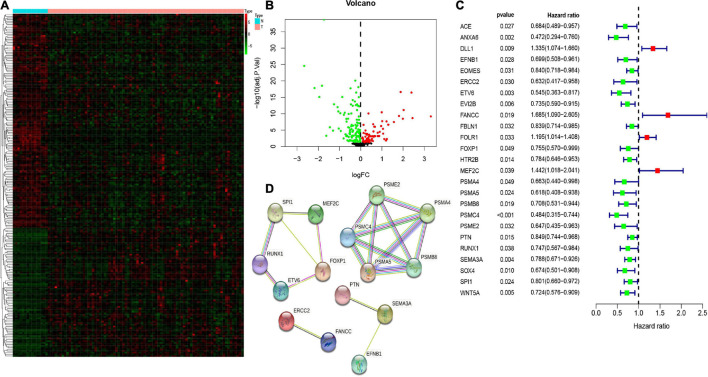
The heatmap **(A)** and volcano **(B)** of differentially expressed genes (DEGs) between osteosarcoma and normal tissues/cells. The univariate Cox regression analysis of 25 prognostic stem cell-related DEGs **(C)**. The protein–protein interaction (PPI) network showed the interactions of 25 prognostic stem cell-related DEGs **(D)**.

Here, we explored the mRNA expression of 413 CSC-related genes in the GSE33383 dataset using the “limma” package in R statistical software. Among the normal and osteosarcoma samples, we used a threshold of FDR value < 0.05 and identified 210 differentially expressed CSC-related genes (Figures 2A,B). Next, we computed the prognostic values of CSC-related DEGs in the TARGET database utilizing the univariate Cox regression analysis. As illustrated in Figure 2C, we observed that altogether, 25 CSC-related genes correlated with the overall survival of osteosarcoma (*p* < 0.05). Hence, the 25 genes were prognostic CSC-related DEGs. Besides, the protein–protein interaction (PPI) network of the 25 prognostic CSC-related DEGs are shown in Figure 2D.

### Establishment and Evaluation of the Prognostic Cancer Stem Cell-Related Gene Signature in the Therapeutically Applicable Research to Generate Effective Treatments

**FIGURE 3:**
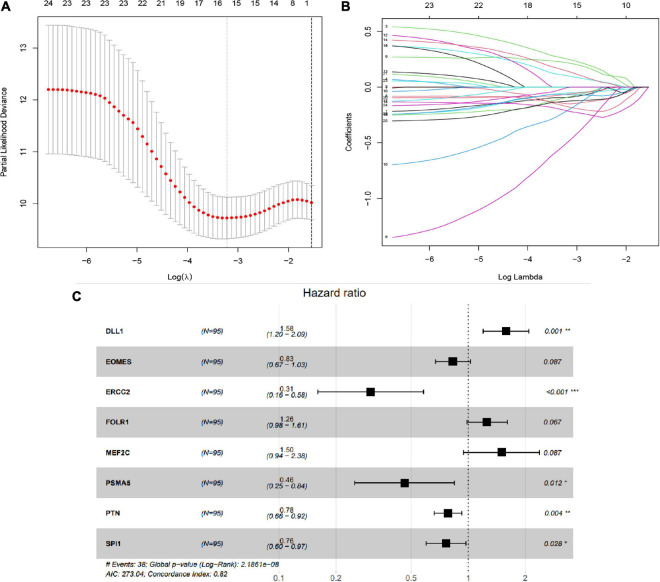
Establishment of stem cell-related gene prognostic signature. **(A,B)** The LASSO Cox analysis determined 15 prognostic genes. **(C)** The forest map of multivariate Cox analysis. *P < 0.05; **P < 0.01; ***P < 0.001.

In this study, we then applied LASSO regression analysis on these 25 CSC-related genes, and 16 genes were selected as candidate genes via relative regression coefficient (Figures 3A,B). Finally, the multivariate Cox analysis was applied, and eight genes [delta-like ligand 1 (DLL1), EOMES, ERCC2, folate receptor 1 (FOLR1), myocyte enhancer factor 2C (MEF2C), PSMA5, PTN, and SPI1] with a minimum Akaike’s information criterion (AIC) value of 273.04 were distinguished to establish the risk signature (Figure 3C). Subsequently, the risk scores were computed on the foundation of the expression level of eight CSC-related prognostic genes, and the corresponding value of coefficient was derived from the multivariate Cox model. Risk score = (0.45792456 × DLL1 expression) + (−0.1851977 × EOMES expression) + (−1.1867301 × ERCC2 expression) + (0.22938571 × FOLR1 expression) + (0.40383058 × MEF2C expression) + (−0.775786 × PSMA5 expression) + (−0.246729 × PTN expression) + (−0.2701665 × SPI1 expression).

**FIGURE 4:**
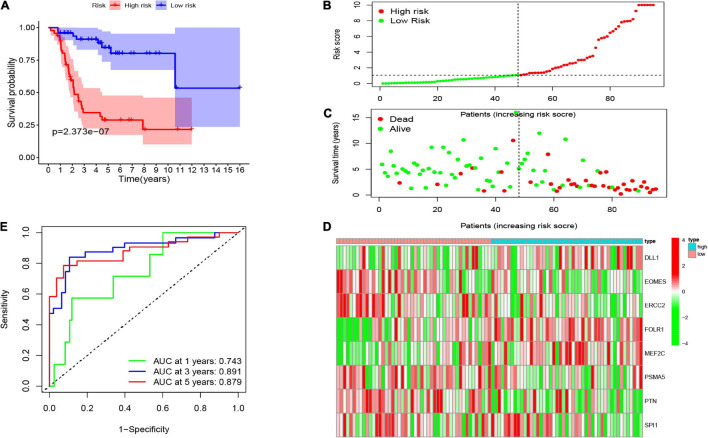
**(A)** The Kaplan–Meier (K-M) curve of the eight-gene signature in the TARGET cohort. The risk score **(B)**, survival time and status **(C)**, as well as the expression of the eight genes **(D)** in the TARGET set. **(E)** The receiver-operating characteristic (ROC) curve and AUC value of the eight-gene signature in the TARGET set.

Consequently, the patients were separated into two risk groups (low and high) on the foundation of the median risk score. As demonstrated in Figure 4A, the K-M curves revealed that the high-risk group had an extremely poorer survival rate than the patients in the low-risk group (*p* = 2.373e–07), showing that the risk score has a valid prognosis value. The risk score and survival status of all osteosarcoma samples were presented in the form of risk plot and scatter, respectively. Moreover, as shown in Figures 4B,C, the mortality and risk coefficient of low-risk patients were lower compared with that of high-risk patients. The assignation of these eight gene expressions in the subgroups of the risk score are shown in Figure 4D. Besides, we used ROC curves to appraise the precision of these eight CSC-related gene signature in forecasting the overall survival of osteosarcoma patients. The AUC values of 1, 3, and 5 years were 0.743, 0.891, and 0.879, respectively (Figure 4E). This shows an outstanding potentiality of our eight CSC-related gene model in predicting.

### Assessment of Eight Cancer Stem Cell-Related Gene Model as Independent Prognostic Factor

**FIGURE 5:**
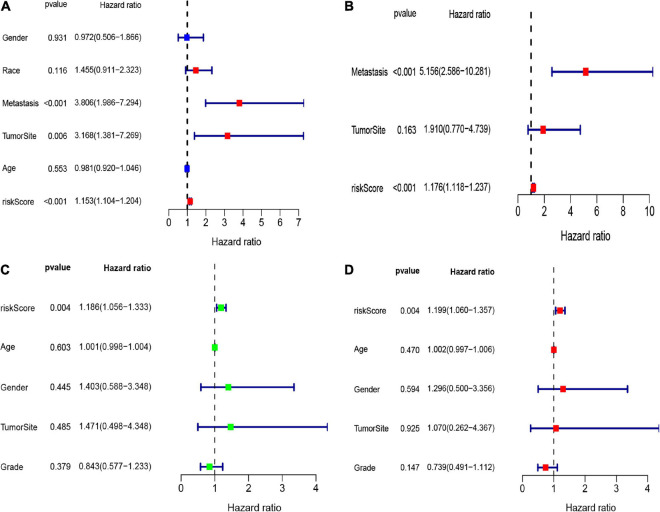
The forest maps of univariate and multivariate Cox regression analysis in the TARGET **(A,B)** and GEO **(C,D)** datasets.

**FIGURE 6:**
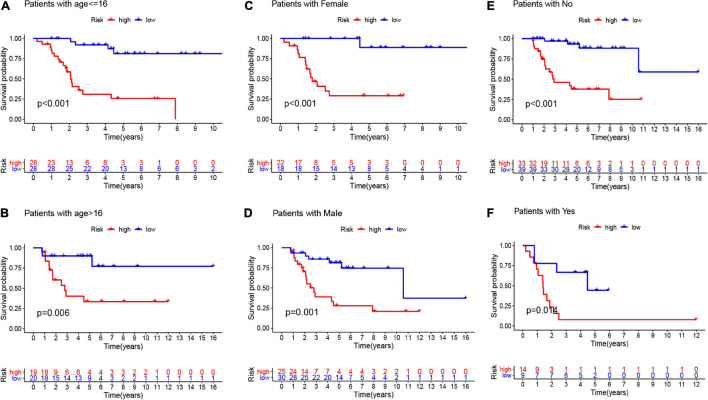
The K-M curves for the two groups (high risk vs. low risk) assigned by clinical factors, comprising age **(A,B)**, gender **(C,D)**, and metastasis **(E,F)**.

Here, we employed Cox regression analysis tests to examine whether the eight CSC-related genes signature was an independent prognostic variable for other clinical traits, like age, gender, race, tumor site, and metastasis. The univariate Cox regression analysis illustrated that the risk score was an independent variable for predicting the prognosis of osteosarcoma patients (HR = 1.157, 95% CI: 1.106-1.211, and *p* < 0.001, Figure 5A). The multivariate Cox regression analysis also showed that the risk score was an independent prognostic factor (*p* < 0.001, HR = 1.191, 95% CI: 1.125-1.260, Figure 5B). Besides, to explore the prognostic value of the signature in osteosarcoma patients sorted by clinical variables, we divided the samples into distinct subgroups according to age, gender, and metastasis. As shown in Figure 6, the overall survival of osteosarcoma patients in the low-risk group was remarkably better than that of the high-risk group (*p* < 0.05).

### Verification of the Risk Signature in the Gene Expression Omnibus Database

**FIGURE 7:**
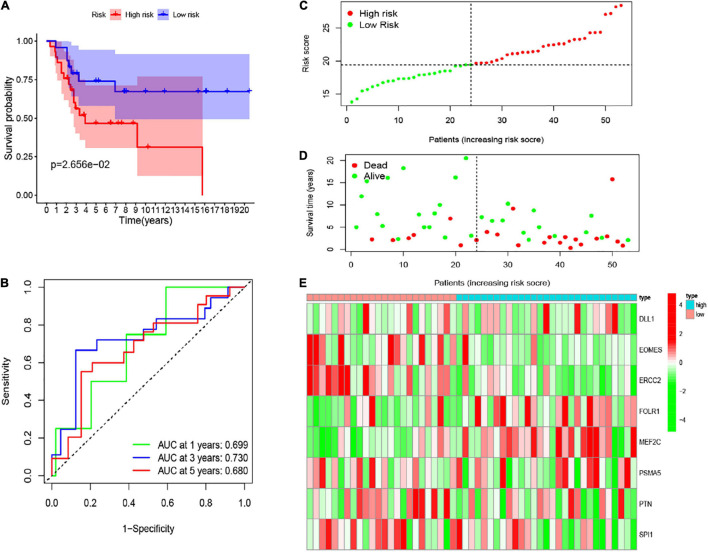
**(A)** The K-M curve of the eight-gene signature in the GEO test cohort. **(B)** The ROC curve and AUC value of the eight-gene signature in the GEO set. **(C–E)** The risk score, survival time, and status as well as the expression of the eight genes in the GEO set.

To validate the robustness of these eight CSC-related gene signature, we employed another independent cohort GSE21257. Based on the formula of the risk score above, the patients of the GSE21257 cohort were assigned into two risk groups, with 29 samples in the high-risk group and 24 samples in the low-risk group. Similar to previous results, the result of the K-M curve also verified that patients in the low-risk group had seriously longer overall survival than those in the high-risk group (Figure 7A). The AUC values of the CSC-related gene signature for predicting the 1-, 3-, and 5-year survival were 0.699, 0.730, and 0.680, respectively (Figure 7B). The heatmap illustrated the gene expression levels of signature in the two risk groups of the validation set. The risk score and survival status of osteosarcoma samples in GSE21257 were also displayed in the form of a risk plot and scatter, respectively (Figures 7C–E). Besides, the univariate (Figure 5C) and multivariate (Figure 5D) Cox analysis explored that the risk score was also an independent prognostic biomarker for patients of osteosarcoma in the GSE21257. All of the results confirmed that the eight CSC-related gene signature was valid and robust.

### Establishment of Nomogram

**FIGURE 8:**
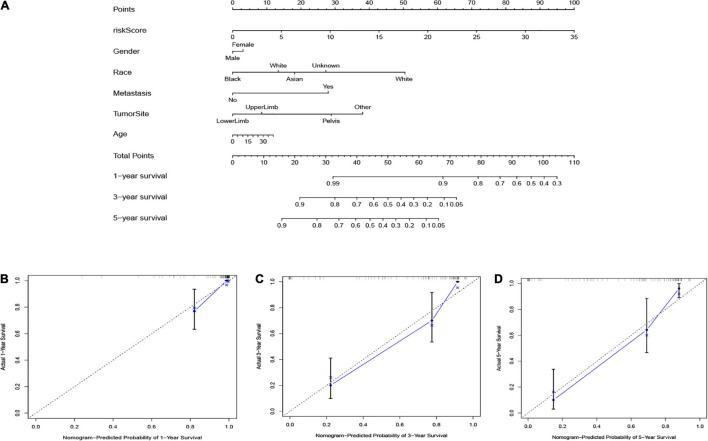
**(A)** The nomogram based on the TARGET database. The calibration curves were applied to predict the 1- **(B)**, 3- **(C)**, and 5-year **(D)** survival in the TARGET cohort.

To forecast the 1-, 3-, and 5-year overall survival of each patient, we performed a nomogram on the foundation of the TARGET dataset. As shown in Figure 8A, the expression signature for risk score, age, gender, race, tumor site, and metastasis were employed as variables. Notably, as demonstrated in Figures 8B–D, the calibration curve of the 1-, 3-, and 5-year overall survival were obtained, and they compared well with the ideal model.

### Risk Score and Potential Biological Functions

**FIGURE 9:**
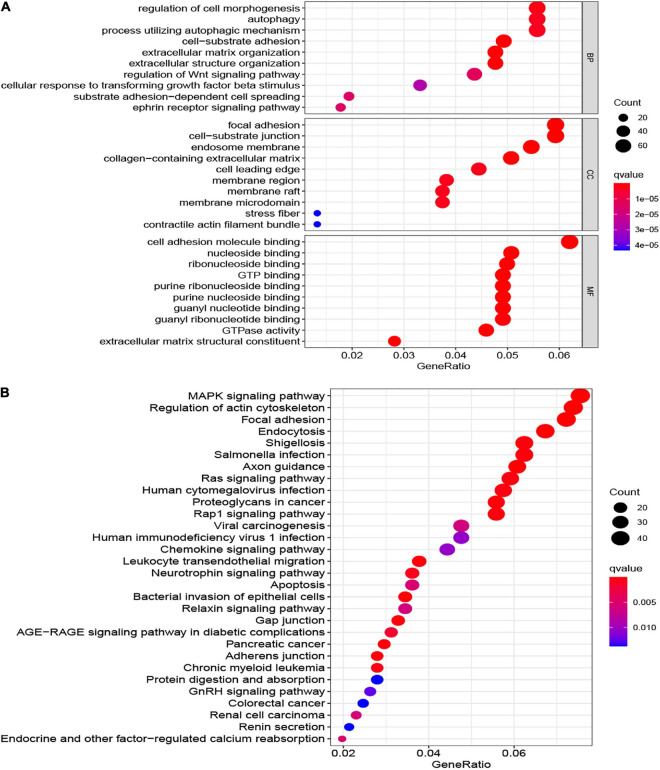
The most significant outcomes of GO **(A)** and KEGG **(B)** analysis of DEGs between high- and low-risk group.

To explore the underlying biological functions between the two risk groups, we identified the DEGs among the two groups. The top 10 GO terms and KEGG of 1,386 DEGs are shown (Figure 9). The most enriched GO terms were CC such as focal adhesion, cell-substrate junction, endosome membrane, collagen-containing extracellular matrix, cell leading edge, and so on; MF, for example, cell adhesion molecule binding, nucleoside binding, ribonucleoside binding, GTP binding, and so on; and BP such as regulation of cell morphogenesis, autophagy, regulation of Wnt signaling pathway, ephrin receptor signaling pathway, and so on (Figure 9A). The KEGG pathway of 1,386 DEGs were mainly related to the Ras signaling pathway, MAPK signaling pathway, chemokine signaling pathway, Rap1 signaling pathway, apoptosis, and so on (Figure 9B).

## Discussion

Osteosarcoma is a common malignant tumor of the bone with an elevated tendency of invasion and metastasis (Chen et al., 2021). Although there are multiple advances in comprehensive therapies for osteosarcoma, the prognosis of osteosarcoma patients is still poor. Thus, exploring the valid prognostic biomarkers and potential therapeutic targets is very critical for improving the prognosis of osteosarcoma. CSCs are a set of heterogeneous cells that own distinct differentiation abilities. Very little subpopulations of cancer cell have properties of stem cell in tumor tissues, but the potential of differentiation and proliferation as well as the abilities of self-renewal are the derived factors for tumorigenesis, development, invasion, recurrence, metastasis, and resistance. In this study, we explored the expression profile of 413 CSC-related genes between normal and osteosarcoma cohorts and obtained 210 genes that were differentially expressed among normal and osteosarcoma tissues in the GEO dataset. Then, we employed the univariate Cox regression analysis to explore the correlation between 210 differentially expressed CSC-related genes and the prognosis of osteosarcoma patients. Finally, an eight CSC-related gene signature was constructed by LASSO and multivariate Cox analysis. The curves of K-M and ROC confirmed that the predictive performance of the signature is stable and consistent with the TARGET internal and GEO external validation sets, and it was remarkably associated with the clinicopathological features and matrix score. What is more, the Cox analyses indicated that the eight-gene signature had an independent predictive capability for prognosis in two distinct cohorts. All of the results showed that the eight-gene signature had a significant potential in clinical application.

In the eight-gene signature identified and validated on the foundation of CSC-related genes, DLL1, FOLR1, and MEF2C were risk variables, while EOMES, ERCC2, PSMA5, PTN, and SPI1 were protective factors. Delta-like ligand 1 was highly expressed in metastatic osteosarcoma cells compared with osteoblasts and non-metastatic osteosarcoma cells (Hughes, 2009). Besides, Pu et al. (2017) found that the expression of DLL1 is negatively associated with multichemoresistance of osteosarcoma. MiR-34a-5p enhances multichemoresistance of osteosarcoma by repressing DLL1. Folate receptor 1 is overexpressed in varieties of solid tumors (Necela et al., 2015; Huang et al., 2018; Kim et al., 2018) and is closely associated with the prognoses of multiple malignant tumors, such as triple-negative breast cancer (Ginter et al., 2017), ovarian cancer (Kobel et al., 2014), and non-small-cell lung cancer (Iwakiri et al., 2008). Myocyte enhancer factor 2C was overexpressed in hepatocellular carcinoma. MEF2C-mediated VEGF-induced vasculogenic mimicry, angiogenesis, and invasion, as well as inhibition of β-catenin-induced tumor growth (Bai et al., 2015). Besides, MEF2C was upregulated in gefitinib-resistant cancer tissues and associated with gefitinib resistance in hepatic cancer cells (Zhang et al., 2018). He et al. (2021) found that EOMES was down expressed in tissue samples and related to the advanced stage and poor prognosis of hepatocellular carcinoma. Moreover, EOMES is associated with the disease-free survival time in gastric cancer (Cui et al., 2020) and also associated with better overall survival in triple-negative breast cancer (Wang et al., 2018). The gene polymorphisms of ERCC2 and ERCC1 were related to chemotherapy sensitivity and overall survival in osteosarcoma patients (Cao et al., 2015). PSMA5 is dysregulated in prostate cancer and associated with bortezomib resistance as well as disease-free survival (Fu et al., 2019). PTN is highly expressed in osteosarcoma, and by target PTN, miR-627-3p inhibits proliferation and migration of osteosarcoma cells (He et al., 2019). LncRNA OIP5-AS1 is related to doxorubicin resistance of osteosarcoma via mediating miR-137-3p/PTN axis (Sun et al., 2020). Besides, the whole transcriptome RNA-seq also proved that PTN is differentially expressed in osteosarcoma (Reimann et al., 2014). Bioinformatics analysis showed that SPI1 was a prognosis-related gene in esophageal squamous cell carcinoma (Ho et al., 2017; Zhang et al., 2020). Most of these genes were related to prognosis in the other cancers, but whether these genes relate to the prognosis of osteosarcoma by altering the molecular mechanism of stem cells still is unclear because only a few correlational researches about these genes have been described.

Next, we assessed the association between risk score, gender, age, and metastasis in osteosarcoma patients through Cox regression analysis. All of the outcomes demonstrated that the risk score could be the independent prognostic variables in patients with osteosarcoma. Besides, based on the risk score, age, gender, race, tumor site, and metastasis, we established a nomogram for forecasting the prognosis of the osteosarcoma patients. From this nomogram, we were able to predict the 1-, 3-, and 5-year survival of osteosarcoma cases. The calibration plots verified that the signature could exactly appraise the survival of the osteosarcoma patients. Finally, in the past years, the underlying mechanisms of tumor susceptibility to stem cells have become a hot topic for research. On the foundation of the DEGs between two risk groups (low and high), we performed GO and KEGG analysis, which demonstrated that several tumor-related biological functions were very affluent, such as the MAPK signaling pathway, Ras signaling pathway, chemokine signaling pathway, and apoptosis.

Despite the many advantages outlined above, our study has some drawbacks. First, there was lack of adequate cases and other clinicopathological information. Second, the performance of our prognostic signature lacked validation in more independent databases. Last, all of our results were on the foundation of the existing opening databases and lacked basic experimental verification as well as clinical information support.

## Conclusion

In summary, we established a new prognostic signature based on eight stem cell-related genes. It proves that it is an independent factor that correlates with overall survival, hence, offering a novel orientation to forecast the prognosis of the osteosarcoma patients.

## Data Availability Statement

The datasets presented in this study can be found in online repositories. The names of the repository/repositories and accession number(s) can be found in the article/supplementary material.

## Author Contributions

BX designed the research study. BX, LL, ZC, YX, and YZ performed the literature search and statistical analysis. BX, AL, YX, PW, and CX interpreted the data and drafted the manuscript. BX and HL critically revised the manuscript. All authors read and approved the final manuscript.

## Conflict of Interest

The authors declare that the research was conducted in the absence of any commercial or financial relationships that could be construed as a potential conflict of interest.

## Publisher’s Note

All claims expressed in this article are solely those of the authors and do not necessarily represent those of their affiliated organizations, or those of the publisher, the editors and the reviewers. Any product that may be evaluated in this article, or claim that may be made by its manufacturer, is not guaranteed or endorsed by the publisher.
